# Impact of a departmental protocol and training on physician confidence in paediatric emergency front of neck access

**DOI:** 10.1097/EA9.0000000000000049

**Published:** 2024-03-04

**Authors:** Éanna O'Sullivan, Bill Walsh

**Affiliations:** From the Department of Anaesthesiology and Pain Medicine, Children's Health Ireland at Temple Street, Dublin (EOS, BW), School of Medicine, University College Dublin (BW), Ireland

Editor,

Emergency front-of-neck access (eFONA) is a potentially life saving procedure recommended by international airway management guidelines in children when oxygenation attempts have failed.^1,2^ In the first instance, this should be performed by an ear, nose and throat surgeon, however one may not be immediately available during this time-critical emergency. There are several approaches described but there is no consensus on the best technique for children.^3^ It has been recommended that every paediatric anaesthesia department should have a locally agreed protocol for this scenario.^4^ Based on the available evidence we devised such an algorithm.^3,5^ Our study aimed to assess whether members of our department had a clear plan for eFONA anatomical target, technique and equipment selection in paediatric patients before the introduction of this protocol and whether their confidence in the appropriateness of their chosen eFONA strategy increased after a structured training session.

This was a prospective observational study in a quaternary paediatric hospital. A departmental algorithm for paediatric eFONA was designed based on the available literature (supplemental figure 1). The algorithm considers the child's age and whether the cricothyroid membrane (CTM) is easily palpable. The “scalpel, bougie, tube” technique was selected as it has the highest reported success rate in infant-sized larynxes.^3,5^ It is also the approach recommended by the Difficult Airway Society for adult eFONA and is thus familiar. The required equipment (one scalpel, four endotracheal tube sizes and two bougie sizes) and a laminated algorithm were presented in an eFONA box which has been added to the difficult airway trolley in both the operating theatre and the intensive care unit. The equipment size is stratified based on age. The target for eFONA, the CTM or the tracheal rings, is determined based on the age of the child and whether the anatomy is easily palpable or not.

A paediatric larynx was 3D-printed using software provided by Duggan et al^6^ (supplementary figure 2). The ‘adult 3d-cric-trainer’ was scaled down, based on CTM height, to that of a 7-year-old larynx using measurements obtained in a previous study.^7^ CTM width has previously been shown to be consistently larger than height and therefore height is the determining factor in tube size selection.

Ethical approval for this study (REC-209-22) was provided by the Children's Health Ireland Research Ethics Committee, Dublin, Ireland (Chairperson Prof. J Browne) on 14 December 2022. Consenting participants (consultants and trainees in paediatric anaesthesia and critical care) were enrolled using convenience sampling. Participants completed a questionnaire immediately before a didactic presentation and practical training session on the proposed eFONA algorithm. The presentation explained the different arms of the algorithm, the procedural details of the proposed eFONA techniques (scalpel, bougie, tube tracheotomy or cricothyroidotomy) as well as the evidence underpinning the algorithm. The practical training session focused on decision-making. The 3D-printed larynx was covered with artificial skin (Gospire), mounted on a wooden spatula, draped and positioned on an operating table (supplemental material). The participant was presented with a clinical scenario necessitating eFONA in a seven-year-old and was asked to proceed as per the algorithm. This provided an opportunity to navigate the algorithm including selection of the appropriate anatomical target, technique, and size of equipment. They then performed the eFONA technique, with success defined as correct placement of the ETT within the airway. They completed an identical questionnaire immediately after the training session and at twelve months. The participants’ confidence levels in the appropriateness of their selected eFONA strategy and familiarity with the equipment required were assessed using a six-point numeric rating scale (1, not at all confident; 6, very confident).

GraphPad Prism Version 9 (GraphPad, USA) was used for analysis. Categorical data was presented as frequency (%). Discrete and continuous data were presented as median [interquartile range]. Between-group comparisons were performed using Fisher's Exact test and Wilcoxon matched-pairs signed rank test as appropriate. The study was conducted between December 2022 and December 2023.

Twenty-seven participants were enrolled, 11 consultants and 16 trainees. Only six (22%) participants had previous training in paediatric eFONA with only two (7.4%) having prior hands-on experience. The median number of prior training sessions in the last 5 years was 0 [0 to 0]. All participants successfully positioned the ETT in the airway. Immediately after training, all 27 (100%) participants had a clear plan for paediatric eFONA strategy compared with 10 (37%) before (*P* < 0.0001). Immediately post training, confidence in the appropriateness of their strategy increased from 2 [1 to 3] before to 4 [3 to 5] after: the median (95% CI) of the before/after difference was 2 (1 to 2), *P* < 0.0001). Familiarity with the equipment required increased from 3 [2 to 4] before to 6 [5 to 6] after: the median (95% CI) before/after difference was 3 (1 to 3), *P* < 0.0001). Although 23 (85.2%) participants were aware that there was an eFONA box only 10 (37%) knew its location: after training these increased to 27 (100%), *P* = 0.11 and *P* < 0.0001 respectively. However, twelve-months after the training only 84% of the respondents still had a clear plan for the paediatric eFONA strategy compared to 100% immediately post training, *P* = 0.047). Similarly, confidence in the appropriateness of their strategy had decreased from 4 [3 to 5] to 3 [2 to 4]: the median (95% CI) reduction was 1 [2 to 0], *P* = 0.01). Familiarity with the equipment had also decreased from 6 [5 to 6] to 5 [4 to 5]: the median (95% CI) reduction was 1 [2 to 0], *P* = 0.01). Finally, at the 12 months follow up only 80% knew the correct location of the eFONA box (*vs.* 100%, *P* = 0.02).

There are multiple different approaches to paediatric eFONA described in the literature.^3^ It is recommended that a standardised approach to this procedure should be agreed upon within a department of paediatric anaesthesia depending on local skillset and available equipment.^4^ The design, teaching and implementation of this algorithm highlighted the importance of having a clear and predetermined plan for the anatomical site, technique, and equipment required for this life-threatening situation. The decision to perform eFONA is difficult, deciding how to perform it should not be. The follow-up questionnaire demonstrated the requirement for regular training.

This study was limited to providing a clear departmental strategy for paediatric eFONA and assessing physicians’ confidence in their strategy and the equipment required. It did not assess technical competence. Performing eFONA in a 3D-printed 7-year-old larynx will not train physicians to be competent in infant eFONA.

In conclusion, introduction of a departmental paediatric eFONA strategy ensures that physicians have a clear plan should this traumatic event occur.

## Supplementary Material

Supplemental Digital Content

## Supplementary Material

Supplemental Digital Content
