# Supplementary figures and images for: Impact of a departmental protocol and training on physician confidence in paediatric emergency front of neck access
Source: Eur J Anaesthesiol Intensive Care. 2024 Mar 4;3(2):e0049. doi: 10.1097/EA9.0000000000000049 (PMC11798368; doi:10.1097/EA9.0000000000000049)

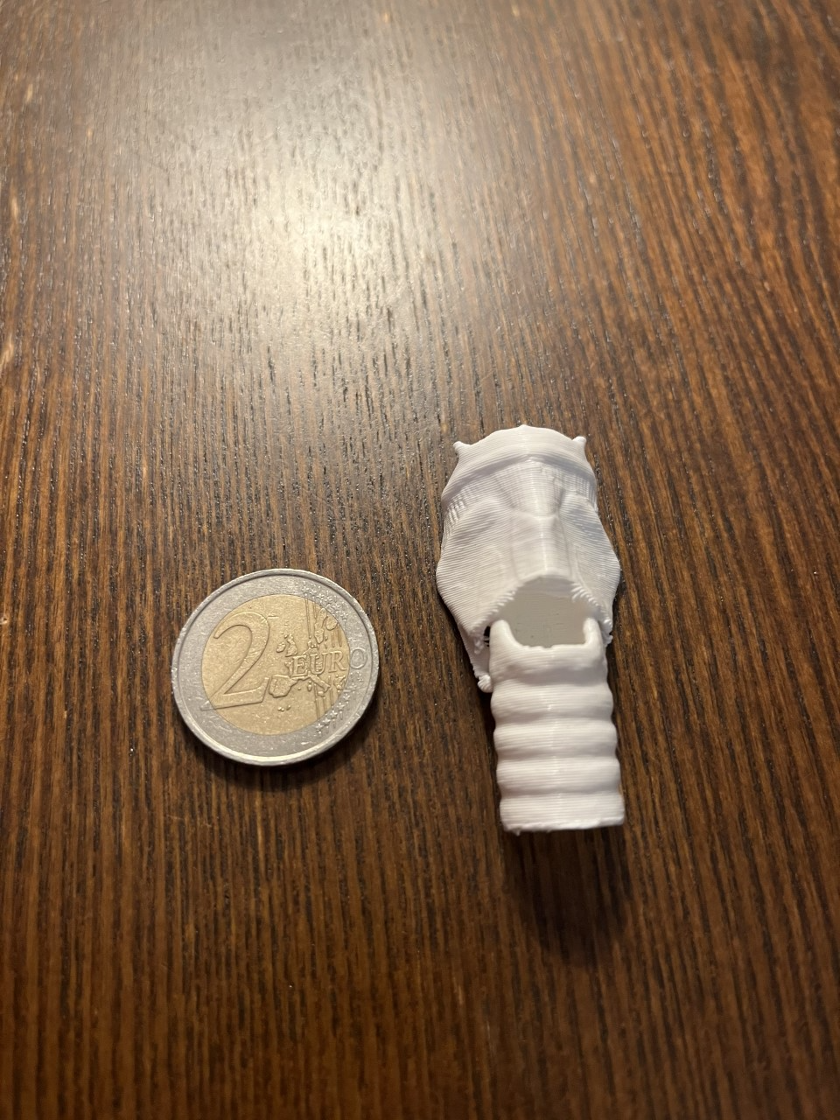

Supplement: Supplemental Digital Content [file ejaic-3-e0049-s001.jpg]
