# Supplementary material for: Impact of a departmental protocol and training on physician confidence in paediatric emergency front of neck access
Source: Eur J Anaesthesiol Intensive Care. 2024 Mar 4;3(2):e0049. doi: 10.1097/EA9.0000000000000049 (PMC11798368; doi:10.1097/EA9.0000000000000049)
Supplement: Supplemental Digital Content [file ejaic-3-e0049-s002.docx]

**Scalpel Bougie Tube (SBT) Technique**^1–11^**:**

Follow this proforma if **ENT IS NOT AVAILABLE** to perform an emergency tracheostomy/rigid bronchoscopy

*CTM = Cricothyroid membrane

2

**Emergency Front of Neck Access (eFONA) Guidelines**

**Contents**

- Bougie size 5 and size 10
- ETT 2.5 (uncuffed)
- ETT size 3, 4, and 5 (Microcuff)
- Scalpel (10 blade)

| **Age** | **Tube** | **Bougie** |
| --- | --- | --- |
| Premature neonate | 2.5 uncuffed | 5 |
| 0-1 yr | 3.0 | 5 |
| 1-7 yr | 4.0 | 10 |
| >7 yr | 5.0 | 10 |

1. Sabato SC, Long E. An institutional approach to the management of the ‘Can’t Intubate, Can’t Oxygenate’ emergency in children. Thomas M, ed. *Paediatr Anaesth*. 2016;26(8):784-793. doi:10.1111/pan.12926

2. Navsa N, Tossel G, Boon JM. Dimensions of the neonatal cricothyroid membrane - How feasible is a surgical cricothyroidotomy? *Paediatr Anaesth*. 2005;15(5):402-406. doi:10.1111/j.1460-9592.2005.01470.x

3. Walsh B, Fennessy P, Ni Mhuircheartaigh R, Snow A, McCarthy KF, McCaul CL. Accuracy of ultrasound in measurement of the pediatric cricothyroid membrane. *Paediatr Anaesth*. 2019;29(7):744-752. doi:10.1111/pan.13658

4. Fennessy P, Walsh B, Laffey JG, McCarthy KF, McCaul CL. Accuracy of pediatric cricothyroid membrane identification by digital palpation and implications for emergency front of neck access. *Paediatr Anaesth*. 2020;30(1):69-77. doi:10.1111/pan.13773

5. CotÉ CJ, Hartnick CJ. Pediatric transtracheal and cricothyrotomy airway devices for emergency use: Which are appropriate for infants and children? *Paediatr Anaesth*. 2009;19(SUPPL. 1):66-76. doi:10.1111/j.1460-9592.2009.02996.x

6. Basaran B, Egilmez AI, Alatas N, Yilbas AA, Sargin M. Accuracy of identifying the cricothyroid membrane in children using palpation. *J Anesth*. 2018;32(5):768-773. doi:10.1007/s00540-018-2538-0

7. Koers L, Janjatovic D, Stevens MF, Preckel B. The emergency paediatric surgical airway: A systematic review. *Eur J Anaesthesiol.* 2018;35(8):558-565. doi:10.1097/EJA.0000000000000813

8. Samuels M, Wieteska S, eds. *Advanced Paediatric Life Support: A Practical Approach to Emergencies*. Sixth Edit. John Wiley & Sons, Ltd; 2016. doi:10.1002/9781119241225

9. WEISS M, ENGELHARDT T. Proposal for the management of the unexpected difficult pediatric airway. *Paediatr Anaesth*. 2010;20(5):454-464. doi:10.1111/j.1460-9592.2010.03284.x

10. Walsh B, Fennessy P, Laffey J, McCarthy K, McCaul C. Paediatric emergency front-of-neck access: accuracy of cricothyroid membrane identification and hypothetical incision lengths. *Br. J. Anaesth.* 2020;125(1):e200-e201. doi:10.1016/j.bja.2020.04.023

11. Prunty SL, Aranda-Palacios A, Heard AM, et al. The “Can’t Intubate Can’t Oxygenate” scenario in pediatric anesthesia: A comparison of the Melker cricothyroidotomy kit with a scalpel bougie technique. *Paediatr Anaesth.* 2015;25(4):400-404. doi:10.1111/pan.12565

12. Riva T, Goerge S, Fuchs A, Greif R, Huber M, Lusardi AC, et al. Emergency front‐of‐neck access in infants: A pragmatic crossover randomized control trial comparing two approaches on a simulated rabbit model. *Paediatr Anaesth*. Published online November 10, 2023. doi:10.1111/pan.14796

13. Ulmer F, Lennertz J, Greif R, Bütikofer L, Theiler L, Riva T. Emergency front of neck access in children: a new learning approach in a rabbit model. *Br J Anaesth*. 2020;125(1):e61-e68. doi:10.1016/j.bja.2019.11.002
